# Serum vitamin B12 levels and 90-day outcomes in hospitalized patients with dementia: A cohort study

**DOI:** 10.1097/MD.0000000000048591

**Published:** 2026-05-01

**Authors:** Meng-Hsun Hsieh, Hsiu-Lan Weng, Kuo-Chuan Hung, I-Chia Teng, Yi-Chen Lai, I-Wen Chen

**Affiliations:** a Department of Anesthesiology, Chi Mei Medical Center, Tainan, Taiwan; b Department of Anesthesiology, E-Da Hospital, I-Shou University, Kaohsiung, Taiwan; c Department of Anesthesiology, Chi Mei Medical Center, Liouying, Tainan, Taiwan.

**Keywords:** biomarker, cobalamin, dementia, mortality, propensity score matching, vitamin B12

## Abstract

Elevated serum vitamin B12 levels have emerged as a paradoxical prognostic marker associated with increased mortality across various clinical settings, yet their significance in hospitalized dementia patients remains unexplored. Given the high vulnerability of this population, we aimed to investigate the association between elevated serum vitamin B12 levels and all-cause mortality. This retrospective cohort study evaluated 90-day outcomes in hospitalized adults with dementia who had serum vitamin B12 levels measured within 7 days of admission (2010–2024). Patients were categorized into exposure (vitamin B12 ≥ 900 pg/mL) and control (300–900 pg/mL) groups. Propensity score matching balanced baseline characteristics, including demographics, comorbidities, and laboratory parameters. The primary endpoint was all-cause mortality within 90 days. Secondary endpoints comprised sepsis, pneumonia, urinary tract infection, and admission to the intensive care unit (ICU). Sensitivity analyses restricted the cohort to patients with unspecified dementia and to patients with a dementia diagnosis established at least 1 year before the index admission. After propensity score matching, 16,513 patients were included in each cohort. Elevated vitamin B12 was significantly associated with increased 90-day mortality (10.9% vs 8.3%; odds ratio [OR] 1.36, 95% confidence interval [CI] 1.26–1.46, *P* < .001), sepsis (4.2% vs 3.0%; OR 1.43, 95% CI 1.27–1.61, *P* < .001), pneumonia (8.3% vs 6.6%; OR 1.28, 95% CI 1.18–1.39, *P* < .001), and ICU admission (3.9% vs 3.0%; OR 1.31, 95% CI 1.16–1.47, *P* < .001). No association was observed with urinary tract infection (OR 0.97, 95% CI 0.90–1.04, *P* = .377). The findings remained robust across sensitivity and subgroup analyses. Elevated vitamin B12 levels are associated with increased short-term mortality, infectious complications, and ICU admission in hospitalized patients with dementia. This readily available biomarker may serve as a potential indicator for risk stratification in this vulnerable population. However, given the observational nature of this study, future prospective studies are warranted to confirm these findings and elucidate the underlying mechanisms.

## 1. Introduction

Globally, dementia is a progressive neurodegenerative condition currently affecting around 57 million individuals, and demographic aging is expected to drive this figure to more than 150 million by 2050.^[1,2]^ Beyond cognitive decline, patients with dementia experience substantial vulnerability during hospitalization due to impaired self-care capacity, communication difficulties, increased susceptibility to delirium, and a high prevalence of multimorbidity.^[3–5]^ Hospitalized dementia patients consistently face significantly elevated mortality rates compared to cognitively intact individuals, with studies reporting in-hospital mortality substantially higher than that of age-matched controls.^[6–10]^ Despite this well-recognized burden, reliable prognostic biomarkers for risk stratification in this vulnerable population remain limited and inconsistently validated. Although multiple prognostic models incorporating clinical variables and laboratory parameters (e.g. serum albumin, urea, and comorbidity indices) have been proposed,^[11,12]^ their applicability in routine practice remains uncertain, underscoring the need for simple and widely available biomarkers to enable early and accurate risk assessment in this vulnerable population.

Among the potential prognostic indicators, elevated serum vitamin B12 has emerged as a paradoxical marker over the past 2 decades. While clinical attention has traditionally focused on vitamin B12 deficiency,^[13–15]^ accumulating evidence demonstrates that high vitamin B12 levels independently predict increased mortality across diverse clinical settings.^[16,17]^ A recent meta-analysis of 22 cohort studies established that each 100 pmol/L increase in serum vitamin B12 was associated with a 4 to 6% higher mortality risk.^[18]^ Notably, most cohorts contributing to this meta-analysis were community or outpatient based with long-term follow-up, with only a minority of hospital-based studies, and none were designed to evaluate short-term in-hospital mortality among patients with dementia.^[18]^ High vitamin B12 levels are believed to reflect underlying pathological processes, including hepatic dysfunction, systemic inflammation, or cellular injury, rather than representing a direct harmful mechanism.^[19–21]^ Given that patients with dementia frequently exhibit chronic low-grade inflammation and multiple organ dysfunction, elevated vitamin B12 may similarly serve as a clinically relevant marker of disease severity in this population.

However, whether elevated vitamin B12 levels retain prognostic significance, specifically in hospitalized patients with dementia, has not been systematically examined. Given the limited availability of validated risk stratification tools in this population, identifying accessible and routinely measured biomarkers could meaningfully inform clinical decision-making, discharge planning, and goal-of-care discussions. Therefore, we conducted a large-scale retrospective cohort study to investigate the association between elevated serum vitamin B12 levels and adverse outcomes among hospitalized adults with dementia.

## 2. Methods

### 2.1. Study design and data source

This retrospective cohort study was conducted using the TriNetX. The database contains information including demographic characteristics, diagnostic codes, procedural records, medication prescriptions, and laboratory measurements. The TriNetX Research Network has been widely utilized in peer-reviewed clinical research across diverse medical disciplines.^[22–25]^ This study aimed to determine whether elevated serum vitamin B12 levels are associated with poor outcomes in adult patients with dementia. Approval for this study was obtained from the Institutional Review Board of the Chi Mei Medical Center. Given that the research involved secondary analysis of fully de-identified electronic health record data, the need for informed consent was waived, consistent with prevailing ethical guidelines for retrospective research.

### 2.2. Study population and cohort definitions

Adult patients aged ≥ 18 years who were hospitalized (acute, non-acute, or general inpatient admissions) (index date) between 2010 and 2024 were considered for inclusion. All patients were required to have a documented diagnosis of dementia at least 1 day prior to admission, including vascular dementia (International Classification of Diseases, 10th Revision [ICD-10] F01), dementia due to other diseases classified elsewhere (F02), or unspecified dementia (F03). Eligible patients must have undergone serum vitamin B12 testing during hospitalization or within 7 days of the index admission. Based on the measured vitamin B12 levels, patients were categorized into exposure group (vitamin B12 ≥ 900 pg/mL) and the control cohort (vitamin B12 300–900 pg/mL).^[17]^ To minimize treatment-related confounding, all included patients were required to have no recorded use of vitamin B12 supplementation (anatomical therapeutic chemical: B03BA) within the 3 months preceding hospitalization.

### 2.3. Exclusion criteria

To reduce confounding from conditions known to profoundly influence vitamin B12 metabolism and short-term mortality risk, patients with a history of toxic liver disease (ICD-10 K71), hepatic failure (K72), human immunodeficiency virus infection (B20), bariatric surgery (Z98.84), end-stage renal disease (N18.6), advanced chronic kidney disease (stage 4–5; N18.4–N18.5), dialysis dependence (Z99.2), or hematologic malignancies (C81–C96) were excluded. In addition, to avoid bias from acute organ failure or severe systemic infection, patients who developed acute kidney injury (N17) or sepsis/severe sepsis (A41 or R65.2) within 1 month prior to the index hospitalization were also excluded from the analysis.

### 2.4. Propensity score matching (PSM)

Propensity score methods were applied to achieve baseline comparability between the exposure and control cohorts. Variables entered into the propensity model encompassed demographic characteristics (age, sex, and race), comorbidities (e.g., essential hypertension), and relevant laboratory measures (including aspartate aminotransferase, alanine aminotransferase, total bilirubin, and C-reactive protein). Patients were matched in a 1:1 ratio using greedy nearest-neighbor matching without replacement, with a caliper set at 0.1 standard deviations of the logit of the propensity score. Balance between groups was assessed using standardized mean differences, with values below 0.1 considered indicative of satisfactory covariate balance. In addition, propensity score distributions were examined visually before and after matching to verify improved overlap and confirm effective mitigation of baseline selection bias.

### 2.5. Outcome definitions and follow-up

The primary outcome was all-cause mortality within 90 days following the index date (i.e., hospitalization), defined by recorded death or an ICD-10 code indicating undetermined cause of death (R99). Secondary outcomes included the occurrence of sepsis or severe sepsis, identified using ICD-10 codes A41 (other sepsis) and R65.2 (severe sepsis); pneumonia, including aspiration pneumonia, captured by ICD-10 codes J12–J18 (viral, bacterial, and unspecified pneumonias) and J69 (pneumonitis due to solids and liquids); urinary tract infection (UTI) defined as N39.0; and intensive care unit (ICU) admission, defined by a record of critical care services (CPT 1013729). All outcomes were assessed within a prespecified follow-up window beginning 7 days after the index date and extending to 90 days. To explore both short-term and longer-term associations, outcomes were additionally evaluated during an early interval (7–30 days) and a late follow-up period (90–365 days) after the index date.

### 2.6. Sensitivity analyses

We performed 2 sensitivity analyses. In Model I, the analysis was restricted to patients with unspecified dementia (ICD-10 F03) to reduce potential heterogeneity arising from different dementia subtypes, which may have distinct underlying pathophysiologies and prognostic trajectories. In Model II, only patients with a documented dementia diagnosis more than 1 year prior to the index hospitalization were included. This restriction was applied to ensure that the dementia diagnosis was well established rather than a recent or incidental finding. For both sensitivity analyses, PSM was performed using the same methodology and covariates as in the primary analysis.

### 2.7. Statistical analysis

Continuous variables were reported as mean ± standard deviation, whereas categorical variables were expressed as frequencies and percentages. Associations between vitamin B12 status and short-term outcomes (7–30 days) as well as overall outcomes during the 7–90-day period were examined using binary regression models, with results presented as ORs and 95% confidence intervals (CIs). For longer-term outcomes assessed from 90 to 365 days after the index date, time-to-event analyses were performed using Cox proportional hazards models to generate hazard ratios (HRs) with corresponding 95% CIs. The proportional hazards assumption was evaluated by inspection of Schoenfeld residuals and statistical testing of time-dependent covariate effects. Prespecified subgroup analyses were conducted by sex (male vs female) and by major comorbidities with baseline prevalence > 20% to maintain analytical validity and statistical power. All statistical analyses were 2-tailed, with *P* values < 0.05 considered indicative of statistical significance.

## 3. Results

### 3.1. Patient selection and baseline characteristics

**Figure 1.**
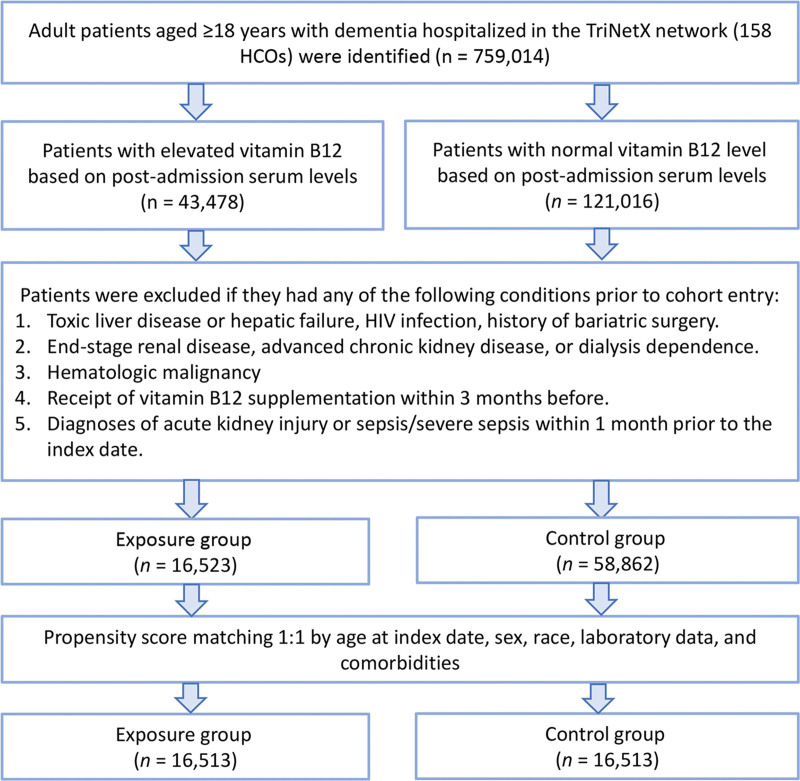
Patient selection flowchart from the TriNetX database. CKD = chronic kidney disease, Control group = vitamin B12 300–900 pg/mL, Exposure group = vitamin B12 ≥ 900 pg/mL, HCOs = healthcare organizations, HIV = human immunodeficiency virus, n = number of patients.

**Figure 2.**
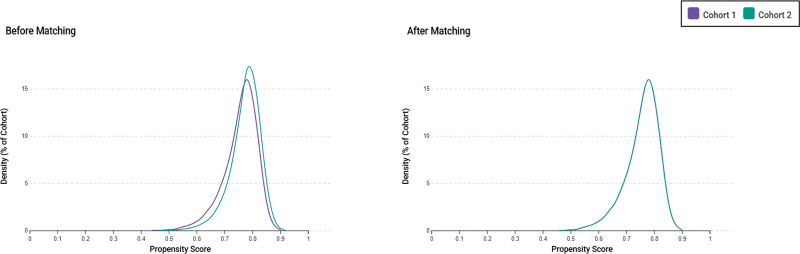
Propensity score density distributions before and after matching. Density plots illustrate the distribution of propensity scores for the exposure group (Cohort 1) and the control group (Cohort 2), demonstrating a substantial baseline imbalance before matching and an improved covariate balance after PSM. Control group = vitamin B12 300–900 pg/mL, Exposure group = vitamin B12 ≥ 900 pg/mL, PSM = propensity score matching.

From the TriNetX database, we identified 759,014 hospitalized adults with a documented diagnosis of dementia. After categorizing patients by vitamin B12 levels and applying the exclusion criteria, 16,523 patients were assigned to the exposure cohort (≥ 900 pg/mL) and 58,862 to the control cohort (300–900 pg/mL). Before PSM, patients in the exposure group were older (79.9 ± 9.4 vs 78.8 ± 10.2 years), more likely to be female (63.3% vs 57.7%), and had a higher prevalence of malnutrition (16.2% vs 10.7%) than controls. Following 1:1 PSM, 16,513 individuals were retained in each group, with baseline characteristics adequately balanced, as all standardized mean differences were < 0.1 (Table 1, Fig. 1). Propensity score density distributions demonstrated substantial improvement in covariate overlap following matching (Fig. 2).

**Table 1:** Baseline characteristics of patients with dementia before and after PSM.

Variables	Before matching			After matching		
	Exposure group (n = 16,523)	Control group (n = 58,862)	SMD†	Exposure group (n = 16,513)	Control group (n = 16,513)	SMD†
Patient characteristics						
Age at index (yr)	79.9 ± 9.4	78.8 ± 10.2	0.119	79.9 ± 9.4	80.1 ± 9.3	0.018
Female	10,458 (63.3)	33,989 (57.7)	0.114	10,450 (63.3)	10,413 (63.1)	0.005
BMI ≥ 30 kg/m^2^	3074 (18.6)	11,922 (20.3)	0.042	3074 (18.6)	3011 (18.2)	0.010
White	11,023 (66.7)	41,376 (70.3)	0.077	11,023 (66.8)	11,090 (67.2)	0.009
Black or African American	2556 (15.5)	7537 (12.8)	0.077	2553 (15.5)	2518 (15.2)	0.006
Unknown Race	1288 (7.8)	5455 (9.3)	0.053	1288 (7.8)	1277 (7.7)	0.002
Asian	1037 (6.3)	2388 (4.1)	0.100	1031 (6.2)	1016 (6.2)	0.004
Comorbidities						
Essential (primary) hypertension	11,325 (68.5)	39,983 (67.9)	0.013	11,318 (68.5)	11,182 (67.7)	0.018
Unspecified dementia	9888 (59.8)	32,271 (54.8)	0.102	9878 (59.8)	9879 (59.8)	0.001
Dyslipidemia	9029 (54.6)	31,593 (53.7)	0.020	9021 (54.6)	8850 (53.6)	0.021
Ischemic heart diseases	5228 (31.6)	18,055 (30.7)	0.021	5227 (31.7)	5112 (31.0)	0.015
Dementia in other diseases classified elsewhere	4765 (28.8)	16,191 (27.5)	0.030	4761 (28.8)	4726 (28.6)	0.005
Diabetes mellitus	4695 (28.4)	16,258 (27.6)	0.018	4692 (28.4)	4588 (27.8)	0.014
Disorders of thyroid gland	4223 (25.6)	13,767 (23.4)	0.050	4217 (25.5)	4116 (24.9)	0.014
Neoplasms	3756 (22.7)	12,516 (21.3)	0.035	3754 (22.7)	3632 (22.0)	0.018
Heart failure	3639 (22.0)	11,282 (19.2)	0.071	3633 (22.0)	3639 (22.0)	0.001
CKD	3051 (18.5)	9273 (15.8)	0.072	3047 (18.5)	3022 (18.3)	0.004
Malnutrition	2675 (16.2)	6279 (10.7)	0.162	2665 (16.1)	2596 (15.7)	0.011
COPD	2356 (14.3)	8220 (14.0)	0.008	2356 (14.3)	2361 (14.3)	0.001
Vitamin D deficiency	2106 (12.7)	7848 (13.3)	0.017	2106 (12.8)	2017 (12.2)	0.016
Vascular dementia	1997 (12.1)	7145 (12.1)	0.002	1997 (12.1)	1956 (11.8)	0.008
Overweight and obesity	1527 (9.2)	5819 (9.9)	0.022	1527 (9.2)	1524 (9.2)	0.001
Nicotine dependence	1234 (7.5)	5600 (9.5)	0.073	1234 (7.5)	1215 (7.4)	0.004
Diseases of liver	1055 (6.4)	3027 (5.1)	0.053	1051 (6.4)	996 (6.0)	0.014
Alcohol related disorders	796 (4.8)	3442 (5.8)	0.046	796 (4.8)	736 (4.5)	0.017
Systemic connective tissue disorders	412 (2.5)	1197 (2.0)	0.031	410 (2.5)	391 (2.4)	0.007
Laboratory data						
Albumin g/dL (≥ 3.5 g/dL)	11,178 (67.7)	40,679 (69.1)	0.031	11,172 (67.7)	11,099 (67.2)	0.009
eGFR ≥ 60 ml/min/1.73m^2^	11,858 (71.8)	41,101 (69.8)	0.043	11,849 (71.8)	11,882 (72.0)	0.004
Hemoglobin A1c ≥ 7%	1634 (9.9)	5760 (9.8)	0.003	1633 (9.9)	1586 (9.6)	0.010
Hemoglobin ≥ 12g/dL	11,719 (70.9)	43,233 (73.4)	0.056	11,716 (71.0)	11,698 (70.8)	0.002
ALT (7–55 U/L)	13,141 (79.5)	46,170 (78.4)	0.027	13,131 (79.5)	13,059 (79.1)	0.011
AST (8–48 U/L)	12,742 (77.1)	44,877 (76.2)	0.021	12,733 (77.1)	12,674 (76.8)	0.008
Total bilirubin (0.3–1.2 mg/dL)	12,427 (75.2)	43,285 (73.5)	0.038	12,418 (75.2)	12,375 (74.9)	0.006
C-reactive protein ≥ 10 mg/L	2052 (12.4)	6570 (11.2)	0.039	2049 (12.4)	2097 (12.7)	0.009
Medication						
Insulins and analogues	3734 (22.6)	12,309 (20.9)	0.041	3727 (22.6)	3684 (22.3)	0.006
ACE inhibitors	3440 (20.8)	13,213 (22.4)	0.040	3440 (20.8)	3379 (20.5)	0.009
Angiotensin II inhibitor	2782 (16.8)	9357 (15.9)	0.025	2778 (16.8)	2742 (16.6)	0.006
Biguanides	1384 (8.4)	5391 (9.2)	0.028	1384 (8.4)	1311 (7.9)	0.016
SGLT2 inhibitors	279 (1.7)	885 (1.5)	0.015	279 (1.7)	281 (1.7)	0.001
GLP-1 analogues	174 (1.1)	603 (1.0)	0.003	174 (1.1)	176 (1.1)	0.001

Data are presented as the mean ± SD or n (%).

ACE = angiotensin-converting enzyme, ALT = alanine aminotransferase, AST = aspartate aminotransferase, BMI = body mass index, CKD = chronic kidney disease, COPD = chronic obstructive pulmonary disease, eGFR = estimated glomerular filtration rate, GLP1 = glucagon-like peptide-1, n = number of patients, SGLT2 = sodium-glucose co-transporter 2, SMD = standardized mean difference..

SMD < 0.1 indicates adequate balance.

### 3.2. Outcomes at 90-day follow-up

At the 90-day follow-up, elevated vitamin B12 levels were significantly associated with higher all-cause mortality compared with normal vitamin B12 levels (OR 1.36, 95% CI 1.26–1.46, *P* < .001) (Table 2). High vitamin B12 levels were also associated with increased risks of sepsis or severe sepsis (OR 1.43, 95% CI 1.27–1.61, *P* < .001), pneumonia (OR 1.28, 95% CI 1.18–1.39, *P* < .001), and ICU admission (OR 1.31, 95% CI 1.16–1.47, *P* < .001). In contrast, no significant association was observed between elevated vitamin B12 levels and UTI (OR 0.97, 95% CI 0.90–1.04, *P* = .377).

**Table 2:** Association between elevated vitamin B12 levels and 90-day outcomes.

Outcomes	Exposure group (N = 16,513) Events (%)	Control group (N = 16,513) Events (%)	OR (95% CI)	*P* value	Risk difference
Mortality	1804 (10.9%)	1366 (8.3%)	1.36 (1.26–1.46)	< .001	2.70%
Sepsis (severe sepsis)	699 (4.2%)	495 (3.0%)	1.43 (1.27–1.61)	< .001	1.20%
Pneumonia	1376 (8.3%)	1095 (6.6%)	1.28 (1.18–1.39)	< .001	1.70%
UTI	1615 (9.8%)	1663 (10.1%)	0.97 (0.90–1.04)	.377	−0.3%
ICU admission	647 (3.9%)	499 (3.0%)	1.31 (1.16–1.47)	< .001	0.90%

CI = confidence interval, control group = vitamin B12 300–900 pg/mL, Exposure group = vitamin B12 ≥ 900 pg/mL, ICU = intensive care unit, N = number of patients, OR = odds ratio, UTI = urinary tract infection.

### 3.3. Outcomes at short- and long-term follow-up

During the early follow-up period (7–30 days), elevated vitamin B12 levels demonstrated stronger associations with adverse outcomes compared with the overall 90-day follow-up, including mortality (OR 1.48, *P* < .001), sepsis (OR 1.52, *P* < .001), pneumonia (OR 1.38, *P* < .001), and ICU admission (OR 1.54, *P* < .001) (Table 3). No significant association was observed with UTI during this early period (OR 1.04, *P* = .469).

**Table 3:** Association between elevated vitamin B12 levels and outcomes at short-term (7–30 days) and long-term (90–365 days) follow-up.

Outcomes	7–30 day		90–365 day	
	OR (95% CI)	*P* value	HR (95% CI)	*P* value
Mortality	1.48 (1.33–1.63)	< .001	1.15 (1.07–1.24)	< .001
Sepsis (severe sepsis)	1.52 (1.29–1.78)	< .001	1.18 (1.06–1.33)	.004
Pneumonia	1.38 (1.24–1.53)	< .001	1.08 (1.00–1.18)	.061
UTI	1.04 (0.94–1.14)	.469	0.98 (0.92–1.05)	.543
ICU admission	1.54 (1.30–1.81)	< .001	1.02 (0.91–1.14)	.700

CI = confidence interval, Control group = 300–900 pg/mL, Expoure group = ≥ 900 pg/mL, HR = hazard ratio, ICU = intensive care unit, OR = odds ratio, UTI = urinary tract infection.

During the extended follow-up period (90–365 days), the associations between elevated vitamin B12 levels and adverse outcomes were attenuated but remained significant for mortality (HR 1.15, *P* < .001) and sepsis (HR 1.18, *P* = .004) (Table 3). In contrast, no significant associations were observed for pneumonia (HR 1.08, *P* = .061), UTI (HR 0.98, *P* = .543), or ICU admission (HR 1.02, *P* = .700). These findings suggest that the prognostic value of elevated vitamin B12 levels is most pronounced in the acute period following hospitalization and diminishes over time for infectious complications and ICU admission, whereas the association with mortality persists into the longer-term follow-up.

### 3.4. Sensitivity analysis

The primary findings remained robust across the sensitivity analyses (Table 4). In Model I, which restricted the analysis to patients with unspecified dementia (ICD-10 F03), elevated vitamin B12 levels were associated with increased 90-day mortality (OR 1.39, *P* < .001), sepsis (OR 1.41, *P* < .001), pneumonia (OR 1.28, *P* < .001), and ICU admission (OR 1.41, *P* < .001). Model II, which included only patients with dementia diagnosed more than 1 year before hospitalization, yielded similar results for mortality (OR 1.45, *P* < .001), sepsis (OR 1.50, *P* < .001), and pneumonia (OR 1.29, *P* < .001). The sensitivity analysis did not demonstrate a significant association with UTI.

**Table 4:** Sensitivity analyses for 90-day outcomes.

Outcomes	Model I	*P* value	Model II	
	OR (95% CI)		OR (95% CI)	*P* value
Mortality	1.39 (1.26–1.55)	< .001	1.45 (1.29–1.63)	< .001
Sepsis (severe sepsis)	1.41 (1.21–1.64)	< .001	1.50 (1.24–1.81)	< .001
Pneumonia	1.28 (1.14–1.44)	< .001	1.29 (1.14–1.47)	< .001
UTI	1.06 (0.96–1.18)	.238	0.99 (0.89–1.10)	.806
ICU admission	1.41 (1.20–1.66)	< .001	1.20 (0.98–1.46)	.077

Model I: restricted to patients with unspecified dementia (ICD-10 F03). Model II: restricted to patients with dementia diagnosed > 1 year before hospitalization.

CI = confidence interval, ICD-10 = International Classification of Diseases, 10th Revision, ICU = intensive care unit, OR = odds ratio, UTI = urinary tract infection.

### 3.5. Subgroup analysis

Subgroup analyses examining 90-day mortality revealed consistent associations across all prespecified subgroups (Table 5). The association between elevated vitamin B12 and mortality was observed in both males (OR 1.43, *P* < .001) and females (OR 1.44, *P* < .001), and this relationship did not differ by sex (*P* for interaction = .957). Similarly, the association persisted regardless of the presence or absence of hypertension, hyperlipidemia, coronary artery disease, diabetes mellitus, thyroid disorders, cancer, or heart failure. These findings suggest that the relationship between elevated vitamin B12 levels and mortality in hospitalized patients with dementia is consistent across diverse clinical subpopulations.

**Table 5:** Subgroup analysis of elevated vitamin B12 and 90-day mortality risk.

Subgroup	OR (95% CI)	*P* value	*P* for interaction
Male	1.43 (1.27–1.62)	< .001	Reference
Female	1.44 (1.30–1.59)	< .001	.957
HTN (+)	1.36 (1.25–1.48)	< .001	Reference
HTN (−)	1.45 (1.24–1.70)	< .001	.518
Dyslipidemia (+)	1.36 (1.24–1.49)	< .001	Reference
Dyslipidemia (−)	1.35 (1.20–1.52)	< .001	.925
CAD (+)	1.37 (1.22–1.54)	< .001	Reference
CAD (−)	1.40 (1.27–1.54)	< .001	.781
DM (+)	1.33 (1.17–1.52)	< .001	Reference
DM (−)	1.45 (1.32–1.59)	< .001	.316
Thyroid disorder (+)	1.37 (1.19–1.56)	< .001	Reference
Thyroid disorder (−)	1.42 (1.29–1.55)	< .001	.697
Cancer (+)	1.41 (1.25–1.59)	< .001	Reference
Cancer (−)	1.31 (1.20–1.44)	< .001	.395
Heart failure (+)	1.38 (1.21–1.57)	< .001	Reference
Heart failure (−)	1.30 (1.19–1.42)	< .001	.505

CAD = coronary artery disease, CI = confidence interval, DM = diabetes mellitus, HTN = hypertension, OR = odds ratio.

## 4. Discussion

In this cohort study of over 33,000 propensity score-matched hospitalized adults with dementia, we found that elevated serum vitamin B12 levels (≥ 900 pg/mL) were significantly associated with increased 90-day all-cause mortality, demonstrating 36% higher observed odds compared with patients with normal vitamin B12 levels. Elevated vitamin B12 levels were also associated with increased risks of sepsis, pneumonia, and ICU admission, but not UTI. These associations remained consistent across sensitivity analyses restricted to unspecified dementia and established dementia diagnoses.

Hospitalized patients with dementia represent a uniquely vulnerable population characterized by high mortality rates that substantially exceed those of cognitively intact individuals.^[7,8,26,27]^ Previous studies have consistently demonstrated that dementia independently increases the risk of in-hospital death, with infection representing one of the leading contributors to mortality in this population. Pneumonia, in particular, has been identified as a predominant cause of death among dementia patients, driven by multiple factors, including dysphagia and aspiration risk, impaired cough reflex, reduced mobility, and compromised immune function.^[28–30]^ Similarly, sepsis carries a disproportionately high mortality burden in this population,^[10,31]^ as patients with dementia often present with atypical symptoms that delay the recognition and treatment of infection. Communication difficulties and altered mental status inherent to dementia further complicate timely diagnosis, while the high prevalence of multimorbidity amplifies physiological vulnerability to infectious insults.

Elevated serum vitamin B12 levels have increasingly been described as part of the so-called “Vitamin B12 paradox,” particularly in elderly and hospitalized populations. Multiple studies have demonstrated that high circulating B12 concentrations are associated with increased mortality.^[16–18]^ Mechanistically, elevated B12 levels are thought to reflect the passive release of cobalamin and its binding proteins from necrotic or injured cells (especially hepatocytes) as well as impaired hepatic clearance in the setting of occult liver dysfunction or systemic inflammation.^[19–21]^ The association between elevated vitamin B12 levels and increased mortality observed in our study is consistent with a growing body of evidence establishing high vitamin B12 levels as prognostic biomarkers across diverse clinical settings.^[16–18]^ A recent meta-analysis of 22 cohort studies demonstrated a 4 to 6% increase in mortality risk per 100 pmol/L increment in serum vitamin B12 levels, supporting a dose-response relationship.^[18]^ Our study extends this evidence in several important ways. First, we specifically examined hospitalized patients with dementia, a population that has not been previously studied in this context.^[18]^ Second, we focused on short-term outcomes within 90 days, whereas most previous studies included in that meta-analysis^[18]^ evaluated long-term mortality over months to years in community-dwelling or outpatient populations. Third, we employed rigorous PSM to control for confounders, including hepatic function, inflammatory markers, and nutritional status. Despite these methodological differences, the consistency of our findings with prior literature strengthens the generalizability of elevated vitamin B12 levels as a mortality predictor.

A novel contribution of our study is the examination of specific infectious outcomes in relation to elevated vitamin B12 levels. While previous research has primarily focused on all-cause mortality, we demonstrated that elevated vitamin B12 levels were significantly associated with increased risks of both sepsis and pneumonia within 90 days of hospitalization. These findings have not been previously reported in patients with dementia and add mechanistic plausibility to the vitamin B12-mortality relationship in this patient population, suggesting that infectious complications may partially mediate the observed mortality risk. The association with pneumonia is particularly relevant given its established role as a leading cause of death in patients with dementia.^[28–30]^ Elevated vitamin B12 levels may identify patients with underlying inflammatory states, nutritional derangements, or subclinical organ dysfunction that predisposes them to both pneumonia development and poor outcomes. Interestingly, we found no association between elevated vitamin B12 levels and UTI at any time point. This divergent pattern implies that the relationship between vitamin B12 and infection is relatively specific rather than reflecting a generalized increase in risk for all infectious complications. The lack of UTI association may reflect the distinct pathophysiology of urinary infections, which are more closely related to anatomical factors, catheter use, and local genitourinary conditions than the systemic inflammatory and metabolic derangements that elevated vitamin B12 levels are thought to reflect.

Elevated vitamin B12 levels were significantly associated with 31% increased odds of ICU admission within 90 days of hospitalization. This finding underscores the potential utility of vitamin B12 as a marker of disease severity and clinical deterioration in hospitalized patients with dementia. ICU admission represents not only a surrogate for acute physiological decompensation but also has substantial implications for patient outcomes, healthcare resource utilization, and goals-of-care decision-making. Notably, while the association between elevated vitamin B12 and ICU admission was robust during the early and overall follow-up (7–90 days) periods, it was attenuated and no longer significant during the extended 90 to 365 days window. This temporal pattern suggests that elevated vitamin B12 primarily identifies patients at risk for acute deterioration requiring intensive care in the short term rather than predicting long-term critical illness. The mechanisms linking elevated vitamin B12 levels to ICU admission likely overlap with those driving mortality and infection risks, including underlying inflammation, hepatic dysfunction, and cellular injury.

Our findings have important clinical implications for the care of hospitalized patients with dementia. The serum vitamin B12 level is a widely available, inexpensive, and routinely measured laboratory parameter. The identification of elevated vitamin B12 levels as an independent factor associated with mortality, infectious complications, and ICU admission suggests that this single biomarker may potentially contribute to risk stratification in this vulnerable population. Clinicians caring for hospitalized dementia patients with elevated vitamin B12 levels may consider closer clinical monitoring, given the observed associations. Furthermore, the ability to identify high-risk patients early in hospitalization could inform resource allocation, discharge planning, and care transitions. Although elevated vitamin B12 levels should not be used in isolation for prognostication, their integration with clinical assessments and other prognostic indicators may warrant further investigation in prospective studies. The development of multivariable prognostic models that integrate vitamin B12 levels with other readily available biomarkers and clinical characteristics represents a promising strategy for enhancing risk stratification and clinical management in this population. Although we applied a predefined threshold of ≥ 900 pg/mL based on prior literature, the optimal prognostic cutoff for risk stratification in hospitalized patients with dementia remains uncertain. Future prospective studies should evaluate whether alternative or higher vitamin B12 thresholds, as well as dose-response relationships across continuous B12 levels, may improve discriminatory performance and enhance positive predictive value. External validation in independent cohorts will be necessary before any specific threshold can be considered for integration into clinical decision-making.

Several limitations warrant consideration. First, as this was a retrospective observational study, causal inference was limited despite rigorous PSM. Residual confounding from unmeasured variables, including frailty status, functional dependence, dementia severity, and baseline cognitive function, cannot be excluded. Second, the TriNetX database lacks granular clinical data, such as dementia staging, behavioral symptoms, feeding difficulties, and detailed nutritional assessments that may influence both vitamin B12 levels and clinical outcomes. Third, vitamin B12 levels were measured at varying times within 7 days of admission, and we could not account for temporal changes, intraindividual variability, or repeated measurements. Although patients with documented vitamin B12 supplementation within 3 months prior to admission were excluded, over-the-counter or externally prescribed supplementation may not have been fully captured. Thus, some elevated B12 levels may reflect unmeasured supplementation rather than underlying disease. While prior studies suggest that markedly elevated B12 in hospitalized elderly patients more often reflects systemic illness than exogenous intake, residual misclassification cannot be excluded and should be considered when interpreting our findings. Fourth, the mechanisms underlying elevated vitamin B12 levels were not directly assessed; we could not distinguish whether high vitamin B12 levels reflected hepatic dysfunction, systemic inflammation, cellular injury, or other pathological processes. Fifth, generalizability may be limited, as TriNetX predominantly captures data from United States healthcare organizations, and the findings may not be applicable to other healthcare settings or populations. Sixth, misclassification of dementia diagnoses based on ICD-10 codes is possible, although sensitivity analyses restricted to established diagnoses yielded consistent results. Finally, we could not ascertain the causes of death or determine whether mortality was directly attributable to the infectious complications identified in our analysis.

## 5. Conclusion

Elevated serum vitamin B12 levels were independently associated with increased short-term mortality, sepsis, pneumonia, and ICU admission among hospitalized adults with dementia. These findings demonstrate statistically significant associations but do not establish causality. This readily available biomarker may represent a marker of underlying disease severity and warrants further prospective investigation before clinical implementation. Future prospective studies should validate these findings, explore the underlying mechanisms linking elevated vitamin B12 to adverse outcomes, and evaluate the integration of vitamin B12 into multivariable prognostic models for hospitalized dementia patients.

## Author contributions

**Conceptualization:** Meng-Hsun Hsieh, Kuo-Chuan Hung, I-Chia Teng, I-Wen Chen.

**Data curation:** Meng-Hsun Hsieh, Kuo-Chuan Hung, I-Chia Teng, I-Wen Chen.

**Formal analysis:** Meng-Hsun Hsieh, Hsiu-Lan Weng, Kuo-Chuan Hung, Yi-Chen Lai, I-Wen Chen.

**Investigation:** Hsiu-Lan Weng, Yi-Chen Lai, I-Wen Chen.

**Methodology:** Hsiu-Lan Weng, I-Chia Teng, I-Wen Chen.

**Validation:** I-Chia Teng, Yi-Chen Lai.

**Writing – original draft:** Meng-Hsun Hsieh, Kuo-Chuan Hung, I-Wen Chen.

**Writing – review & editing:** Meng-Hsun Hsieh, Kuo-Chuan Hung, I-Wen Chen.
